# Risk prediction models for permanent pacemaker implantation following transcatheter aortic valve replacement: a systematic review and meta-analysis

**DOI:** 10.3389/fcvm.2025.1563597

**Published:** 2025-09-25

**Authors:** Yijun Mao, Qiang Liu, Hui Fan, Wenjing He, Xueqian Ouyang, Xiaojuan Wang, Erqing Li, Li Qiu, Huanni Dong

**Affiliations:** ^1^ Department of Nursing, Xianyang Central Hospital, Xianyang, China; ^2^ Department of Orthopedic Surgery, Xianyang Central Hospital, Xianyang, China; ^3^ Interventional Operating Room, Xianyang Central Hospital, Xianyang, China

**Keywords:** conduction disturbance, permanent pacemaker implantation, transcatheter aortic valve replacement, prediction model, systematic review, meta-analysis

## Abstract

**Objective:**

To systematically evaluate the methodological quality and predictive performance of risk prediction models for permanent pacemaker implantation (PPMI) following transcatheter aortic valve replacement (TAVR), identify key predictive factors, and assess the risk of bias and clinical applicability of these models.

**Methods:**

A comprehensive search was conducted across multiple databases, including PubMed, Web of Science, The Cochrane Library, Embase, Cumulative Index to Nursing and Allied Health Literature (CINAHL), China National Knowledge Infrastructure (CNKI), Wanfang Database, China Science and Technology Journal Database (VIP), and SinoMed. The search included all records from database inception to January 1, 2025. Two independent researchers screened studies and extracted relevant data.

**Results:**

A total of 11 studies were included, covering 11 risk prediction models with sample sizes ranging from 184–35,410. The incidence of PPMI after TAVR varied between 7.3% and 31.0%. Frequently identified predictors (present in at least two studies) included right bundle branch block (RBBB), self-expandable valves, PR interval, QRS interval, and atrioventricular block (AVB). All models reported the area under the receiver operating characteristic curve (AUROC), ranging from 0.660–0.916, with seven studies providing calibration metrics. Internal validation was performed in three studies, while one study included both internal and external validation. Ten studies were assessed as having a high risk of bias, primarily due to deficiencies in data analysis. The pooled AUROC for the nine validated models was 0.76 (95% confidence interval: 0.72–0.80), indicating moderate discriminatory ability.

**Conclusion:**

Existing risk prediction models for PPMI after TAVR demonstrate moderate predictive performance but are limited by a high risk of bias, as assessed using the Prediction Model Risk of Bias Assessment Tool (PROBAST). Future research should focus on developing more robust models through larger sample sizes, rigorous methodologies, and multi-center external validation.

**Systematic Review Registration:**

The protocol for this study is registered with https://www.crd.york.ac.uk/PROSPERO/view/CRD42025629869, PROSPERO CRD42025629869.

## 1 Introduction

Aortic valve stenosis is a prevalent valvular heart disease among the older adult, with its incidence increasing with age (1). Transcatheter aortic valve replacement (TAVR) has become the primary treatment for this condition (2). Advances in transcatheter intervention technology, continuous updates in prosthetic valve products, and the growing emphasis on lifelong patient management have expanded the indications for TAVR. Initially limited to very high-risk and high-risk patients, TAVR is now performed on intermediate- and low-risk populations, reflecting a trend toward younger candidates (3). However, long-term postoperative outcomes in TAVR patients have garnered significant attention (4). Despite improvements in technology, devices, and patient care, factors such as advanced age, frailty, and multimorbidity contribute to a high incidence of complications, including cardiac conduction abnormalities and paravalvular leakage (4). Notably, the 1-year readmission and all-cause mortality rates remain high, at 44.2% and 23.7%, respectively (5, 6).

Cardiac conduction block is a serious complication of TAVR, typically occurring within 72 h postoperatively (7). It is closely associated with intraoperative injury to the cardiac conduction system. Procedural factors such as guidewire transvalvular passage, balloon dilation, and valve placement can cause inflammation, edema, or mechanical damage to the conduction system, leading to temporary or permanent conduction block (8). Postoperative conduction block is linked to poor patient outcomes, and its significance is growing as TAVR indications expand to younger and lower-risk populations. A meta-analysis revealed that new-onset conduction block after TAVR increases the risk of cardiovascular events and long-term mortality (9). In cases of heart block, immediate permanent pacemaker implantation (PPMI) is required, with an average PPMI rate of 13% (10–12).

The impact of PPMI on the long-term prognosis of TAVR remains a subject of debate. On one hand, PPMI prevents sudden cardiac death caused by high-grade atrioventricular block (AVB), complete AVB, and bradyarrhythmias. On the other hand, prolonged right ventricular pacing can result in electromechanical dyssynchrony, leading to left ventricular systolic dysfunction (13, 14). While some studies have found no association between PPMI and all-cause mortality or cardiovascular event rates, reporting no significant differences in long-term survival between patients with or without PPMI (15, 16), others have linked PPMI after TAVR to increased risks of 1-year mortality and hospitalization for heart failure (17). Additionally, patients with PPMI, particularly those who were pacemaker-dependent, demonstrated reduced survival rates at 6-year follow-up compared to those without PPMI (18). These conflicting findings may be attributed to inter-study heterogeneity, with variations in baseline population characteristics and surgical risk stratification potentially biasing results.

Risk prediction models estimate the probability of PPMI after TAVR by integrating multiple predictors, including baseline characteristics, computed tomography angiography (CTA) data, electrocardiographic data, echocardiographic data, and procedural factors. Predicting PPMI occurrence allows timely medical intervention, reducing the risk of further complications. Currently, tools such as the European System for Cardiac Operative Risk Evaluation II (EuroSCORE II) (19, 20) and the Society of Thoracic Surgeons Predicted Risk of Mortality (STS-PROM) (21–23) are widely used in clinical practice to evaluate mortality and surgical risk in cardiac surgery patients. However, these traditional surgical risk scores were developed for surgical populations and show limited accuracy in predicting outcomes for TAVR patients (24). Therefore, there is a need for specific risk assessment tools tailored to the characteristics of TAVR patients, particularly for postoperative PPMI. Although the number of risk prediction models for PPMI after TAVR has been increasing, their quality and applicability have not been systematically evaluated. This study aims to identify and critically evaluate published and developed risk prediction models for PPMI in TAVR patients. The findings will provide valuable insights for clinical practice and future research.

## 2 Methods

We applied the PICOTS framework to organize the clinical inquiry (see Supplementary Table S1). The study is registered in the PROSPERO database under the registration number CRD42025629869.

### 2.1 Search strategy

We conducted a comprehensive search across multiple databases from their inception until November 1, 2024, including PubMed, Web of Science, The Cochrane Library, Embase, Cumulative Index to Nursing and Allied Health Literature (CINAHL), China National Knowledge Infrastructure (CNKI), Wanfang Database, China Science and Technology Journal Database (VIP), and SinoMed. The search strategy centered on three key concepts: Transcatheter Aortic Valve Replacement (TAVR), permanent pacemaker implantation (PPMI), and prediction. Detailed search strategies are outlined in Supplementary Table S2.

### 2.2 Inclusion and exclusion criteria

The inclusion criteria were: (1) studies involving patients who underwent TAVR; (2) observational study designs; (3) PPMI after TAVR as the reported outcome; and (4) studies including a predictive model. The exclusion criteria were: (1) studies focusing solely on risk factors for PPMI without developing predictive models; (2) studies without accessible full texts; (3) grey literature, such as conference abstracts and agency reports; (4) duplicate publications; and (5) studies not published in English or Chinese.

### 2.3 Study selection and data extraction

Two reviewers (MY and FH) independently screened the articles according to the inclusion criteria, with a third reviewer (LQ) resolving any disagreements.

Data extracted from the selected studies were classified into four categories: (1) **Basic study information**, including the first author, publication year, study design, data source, study period, and outcome; (2) **Basic model information**, such as sample size, outcome event rate, events per variable (EPV), model development method, variable selection approach, handling of missing data, and processing of continuous variables; (3) **Model performance**, including discrimination, calibration, type of model validation, and formats for presenting prediction models; and (4) **Predictors**, detailing the number of candidate variables and final predictors.

### 2.4 Quality assessment

The risk of bias and applicability of the included studies were assessed using the Prediction model Risk Of Bias ASsessment Tool (PROBAST) (25).

### 2.5 Data synthesis and statistic analysis

A meta-analysis was performed to assess the area under the curve (AUC) of the PPMI prediction model. Review Manager 5.4 software was used to calculate the pooled AUC and its corresponding 95% confidence interval (CI). Heterogeneity among studies was evaluated using the *Q*-test and the *I*^2^ statistic. Substantial heterogeneity was indicated by an *I*^2^ value greater than 50% and a *Q*-test *p*-value ≤ 0.1, prompting the use of a random-effects model. In contrast, low heterogeneity, defined as an *I*^2^ value ≤ 50% and a *Q*-test *p*-value > 0.1, justified the use of a fixed-effects model.

## 3 Results

### 3.1 Study selection

**Figure 1:**
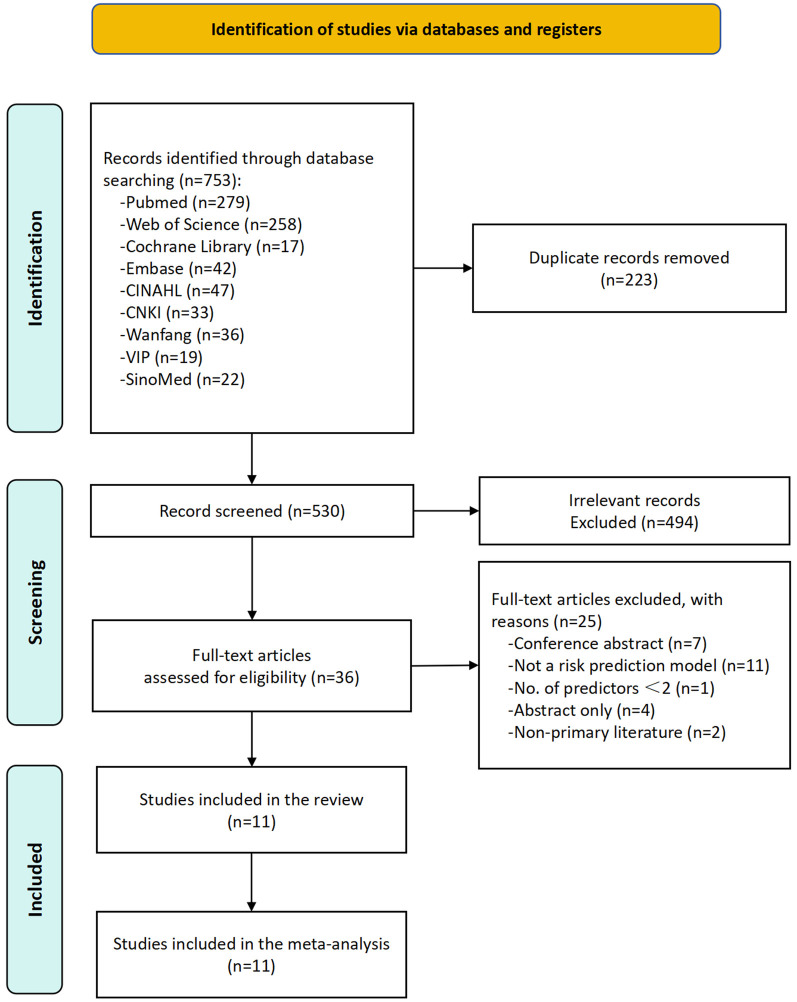
Flowchart depicting a systematic review process. A total of 753 records were identified through database searching. After removing 223 duplicates, 530 records remained for screening. Following screening, 494 records were excluded as irrelevant, and 36 full-text articles were assessed for eligibility. After excluding 25 additional articles (due to reasons such as conference abstracts, non-risk prediction models, fewer than 2 predictors, abstract-only publications, and non-primary literature), 11 studies were ultimately included.

A total of 753 records were identified through database searches, with 223 duplicates removed. After screening the remaining 530 articles, 494 irrelevant records were excluded. An additional 25 articles were excluded for the following reasons: conference abstracts (*n* = 7), absence of a risk prediction model (*n* = 11), fewer than two predictors (*n* = 1), abstract-only publications (*n* = 4), and none-primary literature (*n* = 2). Ultimately, 11 studies were included in the final analysis, collectively reporting 11 prediction models for PPMI following TAVR (Figure 1).

### 3.2 Study characteristics

The basic characteristics of the included studies are summarized in Table 1 and Supplementary Table S3. The studies were published between 2016 and 2024, with nine conducted in the United States and two in China. Of these, nine were retrospective cohort studies, and two were prospective cohort studies. Sample sizes ranged from 184–35,410 participants. The outcomes of interest primarily included pacemaker implantation (*n* = 10), with one study focusing on new-onset conduction disturbances (*n* = 1). Reported PPMI rates ranged from 7.3%–31.0%.

**Table 1:** Basic characteristics of the included studies.

Author (year)	Type	Paper validated	Source data	Region	Patient recruitment years	Main outcome	PPMI cases/sample size (%)	EPV
Agasthi P et al. (26)	D	-	RC	United States of America	2014–2017	PPMI	189/964 (19.6%)^a^	1.29^a^
							176/657 (26.8%)^b^	1.08^b^
Barrett CD et al. (27)	D/V	-	RC	United States of America	2013–2019	PPMI	98/606 (16.2%)	2.72
Black GB 2023	V	Kiani S 2019	PC	United States of America	2019–2020	PPMI	48/661 (7.3%)	NA
Kiani S et al. (28)	D/V	-	RC	United States of America	2013–2018	PMI	87/1,145 (7.6%)	1.98
Liu J et al. (32)	D	-	RC	China	2016–2022	NOCD	57/184 (31.0%)	1.58
Maeno Y et al. (33)	D/V	-	PC	United States of America	2013–2016	PPMI	35/240 (14.6%)	1.13
Qi Y et al. (29)	D/V	-	RC	China	2015–2022	PPMI	54/384 (14.0%)	1.35
Shivamurthy P et al. (30)	V	Vejpongsa P 2018	RC	United States of America	2011–2017	PPMI	90/917 (9.8%)	NA
Truong VT et al. (31)	D	-	RC	United States of America	2011–2019	PPMI	95/557 (17.1%)	2.64
Tsushima T et al. (10)	D/V	-	RC	United States of America	2011–2018	CIED	184/888 (20.7%)	NI
Vejpongsa P et al. (34)	D/V	-	RC	United States of America	2012–2014	PPMI	3,955/35,410 (11.2%)	172.0

CIED, cardiac implantable electronic devices; D, development study; NA, not assessed; NI, no information; NOCD, new-onset conduction disturbance; PC, prospect cohort; PMI, pacemaker implantation; PPMI, permanent pacemaker implantation; RC, retrospective cohort; V, validation study.

^a^ The patients were included for a 30-d analysis.

^b^ The Patients were included for a 1-year analysis.

**Figure 2:**
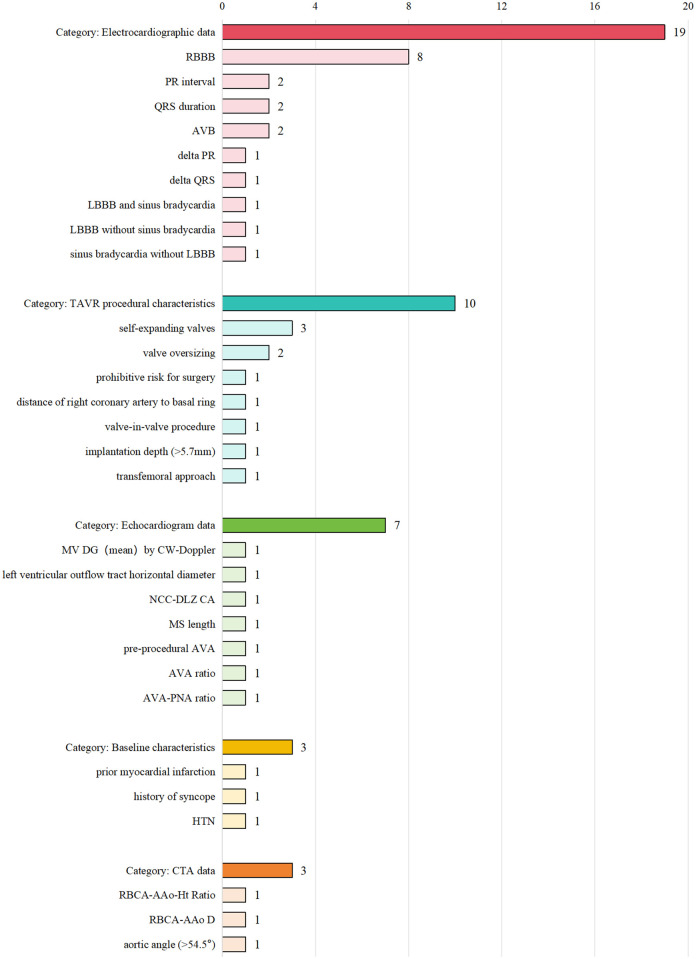
Bar chart illustrating four categories: Electrocardiographic data (19, with RBBB having 8), TAVR procedural characteristics (10, with self-expanding valves having 3), Echocardiogram data (7), Baseline characteristics (3), and CTA data (3). Each category and subcategory is aligned with its respective count.

The model information is presented in Table 2 and Supplementary Table S4. Logistic regression was the primary method used for model development. Additionally, machine learning techniques, such as gradient boosting machine (GBM) and random forest (RF), were applied in some studies. Supplementary Table S5 and Figure 2 summarizes the predictors included in the final models. Right bundle branch block (RBBB) was the most frequently used predictor, appearing in eight models. Other common predictors included self-expandable valve, PR interval, QRS interval, and atrioventricular block (AVB).

**Table 2:** Domains of predictors and performance of PPMI after TAVR risk prediction models.

Author (year)	Missing data handling	Continuous variable processing method	Variable selection	Model development method	Calibration method	Validation method	Final predictors	Model performance	Model presentation
Agasthi P et al. (26)	Eliminate variables with ≥50 missing and near-zero variance; the remaining variables are treated using the GBM algorithm	NI	NI	GBM	NA	5-fold cross validation	Right brachiocephalic artery to aortic annulus distance to patient height ratio	Model 1: A: 0.660 (0.610, 0.700)	NI
							Right brachiocephalic artery to aortic annulus distance		
								Model 2: A: 0.720 (0.670, 0.760)	
							RBBB		
							Self-expanding valves		
							Prohibitive risk for surgery		
Barrett CD et al. (27)	NI	Continuity	NI	Logistic regression model	NA	NA	Prior myocardial infarction	A: 0.804 (0.704, 0.867)	Formula of risk score obtained by regression coefficient of each factor
							RBBB		
							PR interval (>200 ms)		
								B: 0.830 (0.707, 0.953)	
							Self-expandable valve		
							Valve-i*n*-valve procedure		
Black GB 2023	NI	Continuity	NI	Logistic regression model	Hosmer-Lemeshow test	200 bootstrap sampling method		B: 0.810 (0.740, 0.880)	Risk score
Kiani S et al. (28)	NI	Continuity	Backward selection	Multivariable logistic regression model	Hosmer-Lemeshow test	NA	History of syncope	A: 0.729 (0.648, 0.810)	Risk score
							Baseline RBBB		
							Baseline QRS duration (>140 ms)		
								B: 0.778 (0.687,0.870	
							Valve oversizing (≥16%)		
Liu J et al. (32)	NI	Continuity	NI	Logistic regression model	NA	NA	Aortic angle (>54.5°)	A: 0.709 (0.623, 0.795)	NI
							Oversizing ratio (122.9%)		
							implantation depth (>5.7 mm)		
Maeno Y et al. (33)	NI	Continuity	Forward-logistic regression stepwise	Logistic regression model	NA	NA	RBBB	A: 0.916 (0.857, 0.975)	NI
							NCC-DLZ CA		
							MS length		
Qi Y et al. (29)	Multiple imputation	Continuity	Forward and backward selection	Multivariable logistic regression model	NA	5,000 bootstrap sampling method & temporal validation	Prior RBBB	A: 0.700 (0.620, 0.780)	Nomogram model
							Pre-procedural AVA	B1: 0.700 (0.620, 0.790)	
							AVA ratio		
								B2: 0.710 (0.430, 0.990)	
							AVA-PNA ratio		
Shivamurthy P et al. (30)	NI	Continuity	NI	NI	NA	NA		B: 0.674 (0.618, 0.729)	NI
Truong VT et al. (31)	Missing values in data were imputed using a RF algorithm	Continuity	NI	Random forest algorithm; Logistic regression model	Brier score	NA	Baseline RBBB	Model 1: A: 0.810	NI
							PR interval		
							Delta PR	Model 2: A: 0.693	
							QRS interval		
							Delta QRS		
Tsushima T et al. (10)	NI	Continuity	Forward stepwise	Multivariable logistic regression model	NA	NA	HTN	A: 0.780 (0.726, 0.833)	Risk score
							RBBB	B: 0.693 (0.627, 0.759)	
							First-degree AVB		
							Self-expanding valve use		
Vejpongsa P et al. (34)	Direct deletion	Continuity	NI	NI	Hosmer-Lemeshow test	NA	RBBB	B: 0.746 (0.721, 0.772)	Risk score
							LBBB and sinus bradycardia		
							LBBB without sinus bradycardia		
							Sinus bradycardia without LBBB		
							2nd degree AVB		
							Transfemoral approach		

AVA, aortic valve area; AVB, atrioventricular block; AUC, area under the curve; CA, calcium volume; DLZ, device-landing zone; GBM, gradient boosting machine; HTN, hypertension; LBBB, left bundle branch block; MS; membranous septum; NA, not assessed; NCC; noncoronary cusp; NI, no information; PNA, prosthetic nominal area; RBBB, right bundle branch block.

A, development cohort; B, validation cohort; B1, internal validation cohort; B2, external validation cohort.

We considered AUC = 0.5–0.7 as poor discrimination, 0.7–0.8 as moderate discrimination, 0.8–0.9 as good discrimination, and 0.9–1.0 as excellent discrimination.

Model discrimination was reported in all studies, with C-statistic values ranging from 0.660–0.916. Calibration was assessed in seven studies, most commonly using the Hosmer-Lemeshow test.

### 3.3 Surgical characteristics

We conducted a comprehensive analysis of procedural and technical factors influencing the risk of PPM following TAVR. This included surgical technique parameters, baseline anatomical features, procedural specifics, and other potential confounding variables (detailed in Supplementary Table S6). We also examined sources of heterogeneity across studies.

#### 3.3.1 Vascular access approach

The transfemoral (TF) approach was the most frequently used in both PPM and non-PPM groups, although it was slightly more common in the PPM cohort, potentially due to its preferential use in high-risk patients with compromised vascular conditions. The transapical (TA) approach was more frequently employed in the PPM group, likely due to its anatomical proximity to the conduction system, particularly the left bundle branch (LBB). Alternative access routes (e.g., transaortic, subclavian) may influence PPM risk differently due to varying degrees of mechanical stress on the conduction pathways. Overall, the TF approach is generally associated with a lower risk of PPM, while the TA approach may increase the likelihood of conduction system injury due to direct ventricular manipulation. Substantial variability in access strategies across studies may contribute to the observed differences in PPM incidence.

#### 3.3.2 Valve type

Self-expanding valves were associated with a significantly higher incidence of PPM compared to balloon-expandable valves. This may be attributed to their greater radial force and deeper implantation depth, both of which can increase compression on the LBB. Differences in the distribution of valve types across studies (e.g., 87.4% self-expanding in Liu J vs. 17.3% in Agasthi P) likely contributed to inter-study heterogeneity in PPM rates.

#### 3.3.3 Implantation depth

Greater implantation depth was correlated with an increased risk of PPM, possibly due to enhanced mechanical stress on the His bundle and LBB. Variability in how implantation depth was defined (e.g., from the valve's lower edge to the aortic annulus vs. ventricular extension length) may further explain heterogeneity in reported outcomes.

#### 3.3.4 Oversizing

Significant valve oversizing (≥16%) was linked to a higher risk of PPM (41.7% vs. 24.1%), likely due to increased mechanical pressure on the conduction system. Inconsistencies in oversizing calculation methods (diameter-based vs. area-based) across studies represent an additional source of heterogeneity.

#### 3.3.5 Balloon dilation

Pre-dilation may increase the risk of conduction system damage, although the evidence remains inconclusive. Post-dilation was slightly more common in the PPM group (8.6% vs. 7.3%), though the sample size was limited.

#### 3.3.6 Anesthesia method

The choice between general anesthesia and conscious sedation had minimal impact on the risk of PPM.

#### 3.3.7 Baseline conduction abnormalities

RBBB was significantly more prevalent in the PPM group (29%–60% vs. 2.7%–12.2%). First-degree AVB was also more frequent in this cohort (27.6%–44.2% vs. 3.8%–27.9%).

#### 3.3.8 Calcification burden

Patients in the PPM group exhibited higher calcium scores (2,389.97 vs. 2,142.8), suggesting a possible association between calcification burden and increased PPM risk.

#### 3.3.9 Key heterogeneity sources

Valve prosthesis type was the primary contributor to heterogeneity, with additional factors including implantation depth, vascular access route, oversizing practices, and pre-existing conduction abnormalities.

### 3.4 Models validation

Three studies conducted internal validation: two used bootstrapping, and one applied cross-validation. Furthermore, one study performed both internal and external validation.

### 3.5 Results of quality assessment

**Figure 3:**
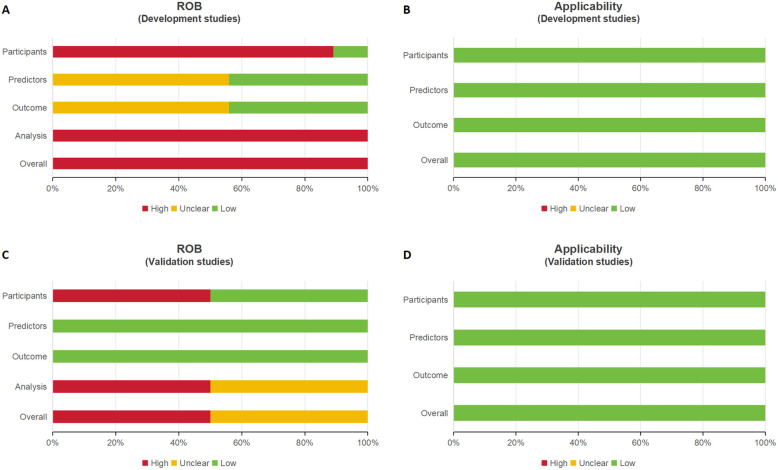
Four bar charts labeled **A** to **D** compare risk of bias (ROB) and applicability in development and validation studies. **A** and **C** assess ROB, showing varying levels of risk with red, yellow, and green bars. **B** and **D** assess applicability, showing predominantly low risk with green bars.

Supplementary Table S7 and Figure 3 provide a summary of the risk of bias and applicability assessments for the included studies. All nine model development studies were judged to have a high risk of bias (Figure 3A) but were considered to have low concerns regarding applicability (Figure 3B). Of the two model validation studies, one was assessed as having a high risk of bias, while the other was rated as having an unclear risk (Figure 3C); both were deemed to have low concerns regarding applicability (Figure 3D).

In the “**participants**” domain, nine studies were assessed as having a high risk of bias, primarily due to the use of inappropriate data sources (10, 26–33). In the “**predictors**” domain, five studies exhibited an unclear risk of bias, as they did not report quality control measures for predictor assessment, likely due to their retrospective design (26, 28, 29, 31, 32). In the “**outcome**” domain, five studies were classified as having an unclear risk of bias because they failed to report whether outcomes and predictors were assessed independently (i.e., blinded assessment) (26, 28, 29, 31, 32).

In the “**analysis**” domain, ten studies were judged to have a high risk of bias due to the following issues:

- Seven studies had insufficient sample sizes, failing to meet the criterion of more than 20 events per variable (EPV) (26–29, 31–33).
- Three studies relied solely on univariate analysis for variable selection (10, 32, 33).
- Seven studies did not comprehensively evaluate the predictive performance of their models (10, 26, 27, 29, 30, 32, 33).
- Six study failed to address model overfitting, underfitting, and optimism in model performance (10, 27, 28, 31, 33, 34).
- Four studies did not report the coefficients of predictors in the multivariate regression model (10, 31–34).
- None of the studies provided details about complexities in the data.

Despite the risks of bias, all eleven studies were assessed as having a low risk of applicability.

### 3.6 Meta-analysis of validation models included in the review

This study conducted a meta-analysis to quantitatively synthesized relevant factors from 11 studies. The meta-analysis identified the following factors as significantly influencing PPMI after TAVR: right bundle branch block (RBBB), self-expandable valve and atrioventricular block (AVB) (Table 3).

**Table 3:** Results of the meta-analysis on PPMI after TAVR predictive factors.

Predictors	No studies	Heterogeneity test		Effects models	Meta-analysis		
		*I*^2^ (%)	*P*		*OR* (95% *CI*)	*Z*	*P*
RBBB	6	71	0.004	Random effects models	8.40 (4.91, 14.37)	7.77	<0.001
Self-expandable valve	2	0	0.660	Fixed effects models	3.57 (2.32, 5.50)	5.79	<0.001
AVB	2	89	0.002	Random effects models	5.42 (1.44, 20.38)	2.50	0.01

Sensitivity analyses were performed using both fixed-effects and random-effects models. The combined effect sizes of the three predictors, along with their corresponding 95% CI, were calculated under both models, revealing no significant differences. These findings indicate that the combined outcomes are highly stable (Table 4).

**Table 4:** Results of sensitivity analyses on PPMI after TAVR predictive factors.

Predictors	No studies	Fixed effects models		Random effects models	
		*OR* (95% *CI*)	*P*	*OR* (95% *CI*)	*P*
RBBB	6	6.89 (5.30, 8.95)	<0.001	8.40 (4.91, 14.37)	<0.001
Self-expandable valve	2	3.57 (2.32, 5.50)	<0.001	3.57 (2.32, 5.50)	<0.001
AVB	2	5.42 (3.52, 8.32)	<0.001	5.42 (1.44, 20.38)	0.01

**Figure 4:**
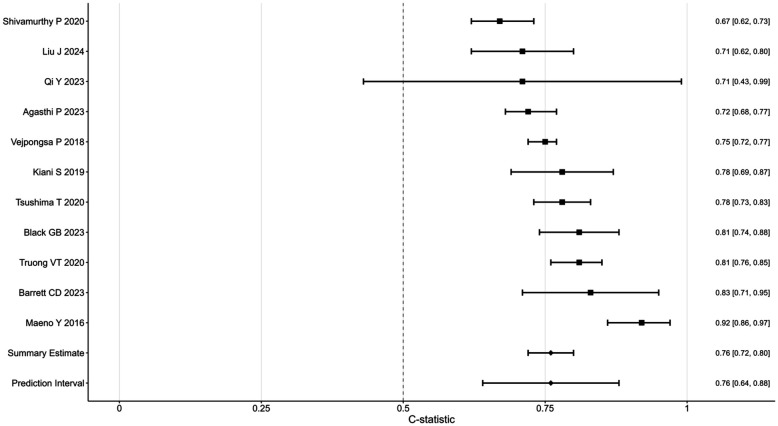
Forest plot showing C-statistics for various studies, each represented by a square with horizontal lines indicating confidence intervals. Studies include Shivamurthy et al. (30), Liu et al. (32), and others, with values ranging from 0.67 to 0.92. Summary estimate and prediction interval appear at the bottom, both at 0.76.

Figure 4 presents a forest plot summarizing the pooled AUC estimates for the predictive performance of the model. Each of the 11 included studies is represented by a square (AUC estimate) and a horizontal line (95% confidence interval), with the size of the square indicating the study's weight in the pooled analysis. The overall pooled AUC was 0.76 (95% CI: 0.72–0.80), suggesting moderate predictive performance. However, substantial heterogeneity was observed (*I*^2^ = 80%, *p* < 0.001), prompting subgroup analyses to explore potential sources of variability in PPMI following TAVR (see Table 5).

**Table 5:** Results of heterogeneity analyses on PPMI after TAVR.

Predictors	Subgroups	No studies	Heterogeneity test		Effects models	Meta-analysis		
			*I*^2^ (%)	*P*		*OR* (95% *CI*)	*Z*	*P*
Publication data	2016–2020	6	90	<0.001	Random effects models	0.78 (0.71, 0.85)	5.27	<0.001
	2021–2024	5	37	0.18	Fixed effects models	0.75 (0.72, 0.79)	11.35	<0.001
Study region	Americas	9	84	<0.001	Random effects models	0.77 (0.75, 0.79)	22.22	<0.001
	Asian	2	0	1.00	Fixed effects models	0.71 (0.63, 0.80)	5.38	<0.001
Valve type	Balloon expandable valve	3	74	0.02	Random effects models	0.84 (0.76, 0.93)	3.31	<0.001
	Self-expanding valve	2	0	1.00	Fixed effects models	0.71 (0.63, 0.80)	5.38	<0.001

#### 3.6.1 Subgroup analysis by publication year

Among studies published from 2016–2020 (*n* = 6), heterogeneity was high (*I*^2^ = 90%, *p* < 0.001), with a random-effects model yielding a pooled odds ratio (OR) of 0.78 (95% CI: 0.71–0.85, Z = 5.27, *p* < 0.001). In contrast, studies published between 2021 and 2024 (*n* = 5) showed lower heterogeneity (*I*^2^ = 37%, *p* = 0.18), and a fixed-effects model produced a pooled OR of 0.75 (95% CI: 0.72–0.79, Z = 11.35, *p* < 0.001). These results suggest greater consistency in model performance in more recent studies.

#### 3.6.2 Subgroup analysis by study region

Studies conducted in the Americas (*n* = 9) demonstrated substantial heterogeneity (*I*^2^ = 84%, *p* < 0.001), with a pooled OR of 0.77 (95% CI: 0.75–0.79, Z = 22.22, *p* < 0.001) using a random-effects model. In contrast, studies from Asia (*n* = 2) showed no heterogeneity (*I*^2^ = 0%, *p* = 1.00), and the fixed-effects model yielded a pooled OR of 0.71 (95% CI: 0.63–0.80, Z = 5.38, *p* < 0.001). These regional differences may reflect variations in patient populations or clinical practices.

#### 3.6.3 Subgroup analysis by valve type

For studies examining balloon-expandable valves (*n* = 3), moderate heterogeneity was observed (*I*^2^ = 74%, *p* = 0.02), with a pooled OR of 0.84 (95% CI: 0.76–0.93, Z = 3.31, *p* < 0.001) from a random-effects model. Studies evaluating self-expanding valves (*n* = 2) exhibited no heterogeneity (*I*^2^ = 0%, *p* = 1.00), and the fixed-effects model estimated a pooled OR of 0.71 (95% CI: 0.63–0.80, Z = 5.38, *p* < 0.001). These findings suggest that valve type may influence the predictive performance of PPMI models.

#### 3.6.4 Interpretation

The high heterogeneity among earlier studies may reflect greater variability in methodological approaches or patient selection criteria. In contrast, the lower heterogeneity in recent studies likely reflects increased standardization in model development and validation. Regional disparities may be attributed to genetic, demographic, or healthcare system differences. The lack of heterogeneity in Asian studies could result from smaller sample sizes or more homogeneous populations. Differences in valve type performance suggest that model accuracy may be influenced by procedural characteristics. Notably, balloon-expandable valves were associated with greater variability, potentially due to heterogeneity in implantation techniques or patient profiles, whereas self-expanding valves showed more consistent outcomes.

These findings highlight the importance of accounting for temporal, geographic, and procedural factors in the development and application of risk prediction models for PPMI after TAVR. Further studies are warranted to better understand sources of heterogeneity and enhance the generalizability of predictive models.

## 4 Discussion

### 4.1 Model performance and quality analysis of study

We evaluated 11 predictive models, all of which demonstrated moderate to good predictive performance during internal or external validation, except for the study by Shivamurthy et al. Reported AUC values ranged from 0.674–0.916. However, based on the PROBAST checklist, ten studies were classified as having a high risk of bias, which limits the generalizability of these predictive models. The pooled AUC for the 11 models included in the meta-analysis was 0.76 (95% CI: 0.72–0.80). The substantial heterogeneity among the studies may be attributed to differences in populations, predictors, and methodologies.

Despite variability in performance and quality, these predictive models provide valuable insights for future research. For example, the study by Barrett et al. utilized randomized blinded number generation to divide patients into training and validation cohorts, a method that ensures the assessor is unaware of predictor information when determining outcomes, thereby preventing observer bias caused by subjective judgment. In contrast, the study by Qi et al. faced challenges, including a sample size yielding an EPV ratio below 20 and data derived from a single-center retrospective design in East China, leading to risks of bias in the participants, predictors, and outcome domains. However, Qi et al. excelled in the analysis domain by addressing missing data through multiple imputation and conducting both internal and external validation to enhance the accuracy and reliability of the model. Many of the included studies were retrospective, which limits the ability to establish causal relationships and highlights the need for prospective validation in external cohorts. Additionally some models relied on univariate analysis for predictor screening, a methodological limitation that should be addressed in future research to improve model robustness and generalizability.

The study by Truong et al. integrated traditional logistic regression with machine learning methods for model construction. Research indicates that machine learning techniques exhibited greater accuracy compared to traditional logistic regression analysis. Research indicates that machine learning outperforms traditional methods in its ability to model complex nonlinear relationships, thereby enhancing predictive accuracy and robustness. Machine learning is particularly effective in managing large-scale, high-dimensional, and incomplete datasets, while also enabling continuous updates as new data become available, thereby improving adaptability and performance. Nonetheless, traditional methods such as logistic regression retain complementary value in certain contexts. The choice of model should depend on factors such as the problem's nature, dataset scale, and data quality.

Overall, while the included models demonstrated moderate to good performance, the high risk of bias highlights the need for significant improvements. Future research should prioritize optimizing sample sizes, implementing robust methods for handling missing data, refining predictor selection processes, accounting for data complexity, and enhancing model fitting techniques.

### 4.2 The predictors used in prediction model

The frequently identified predictors carry significant implications for nursing practice and future research.

Baseline RBBB was identified as the strongest predictor of PPMI after TAVR (35, 36). Studies have shown that baseline RBBB significantly increases the incidence of PPMI after TAVR (37, 38), with patients presenting with preoperative RBBB having nearly a five-fold increased risk of requiring PPMI post-TAVR (39).

Self-expanding valves were also identified as a predictor of PPMI after TAVR (40). The incidence of PPMI was higher in patients receiving self-expanding valves compared to those with balloon-expandable valves (17.4% vs. 6.5%). This discrepancy may be attributed to the unique characteristics of self-expanding valves, including their high radial support and self-expanding properties. These valves have a taller frame, are positioned deeper in the left ventricular outflow tract, and exert continuous pressure on the adjacent conduction system after placement, resulting in a significantly higher rate of PPMI compared to balloon-expandable valves (8).

Oversized valves are associated with an increased risk of PPMI following TAVR (41). Regarding frame morphology, the inflow tract of oversized valves exhibits greater deformation, as the diameter at the lower end of the inflow tract exceeds that at the site of contact with the aortic annulus and leaflets. For instance, the Evolut R 26 mm valve, with its relatively cylindrical inflow tract, is more prone to developing a cratered inflow tract, while the Evolut 34 mm XL valve provides a more stable anchorage site. However, oversized valves are more prone to unpredictable positional self-adjustments after deployment due to uneven depth beneath the annulus, potentially leading to complications such as displacement (42).

Electrocardiographic changes, including QRS widening and PR interval prolongation following TAVR, may indicate damage to the conduction system below the atrioventricular (AV) node and the His bundle. Notably, 82% of patients with a prolonged PR interval after TAVR exhibit a new-onset prolongation of the His-ventricle (HV) interval (43). A prolonged HV interval (>70 ms) increases the risk of AV block by fourfold (44). Based on these findings, electrophysiological testing is recommended for high-risk patients following TAVR to identify potential delayed-onset AV block (45).

While RBBB and valve type emerged as dominant predictors in our analysis, other clinically relevant factors—such as body surface area (BSA), sex, and aortic valve calcium scores—were less frequently incorporated. This likely reflects limitations in the original studies:

• Anatomical factors [e.g., left ventricular outflow tract (LVOT) calcium distribution] were infrequently reported, despite their established association with conduction disturbances (46, 47).
• BSA and female sex have been associated with a higher incidence of conduction abnormalities following TAVR (48–50); however, none of the models included in this review incorporated these variables in their final predictive algorithms.
• Calcium scoring variability, such as differences between Agatston and volume-based methods, may have hindered comparability across studies (51).

To enhance model performance and generalizability, future predictive models should prioritize the standardized collection and reporting of these variables.

## 5 Limitations

Several limitations of this study should be acknowledged. First, some prediction models lacked external validation, thereby limiting the assessment of their generalizability. Second, certain predictive factors were reported in only a single study and thus could not be included in the meta-analysis, potentially influencing the overall results. Third, due to language restrictions, this review included only studies published in English and Chinese, which may have excluded relevant research in other major languages. Fourth, the validation cohorts were predominantly from U.S. populations (9 of 11 studies), with only two based on Chinese cohorts. This geographic imbalance may reduce the applicability of findings to other ethnic groups, particularly Asian populations, where anatomical characteristics of the aortic valve and pacemaker implantation thresholds may differ. Additionally, there remains a paucity of high-quality prospective studies on PPMI prediction models. Given the ongoing evolution of TAVR technology, variations in implantation techniques and patient selection across time may further compromise study comparability.

## 6 Conclusion

This systematic review included 11 studies reporting 11 prediction models for PPMI after TAVR. The results indicated that the pooled AUC for the nine validated models was 0.76 (95% CI: 0.72–0.80), reflecting a moderate level of discriminatory ability. However, most of the included studies were assessed as having a high risk of bias according to the PROBAST checklist. Current prediction models for PPMI after TAVR do not meet PROBAST standards.

To improve the quality of future research, it is essential for researchers to familiarize themselves with the PROBAST checklist and adhere to the reporting guidelines outlined in the Transparent Reporting of a Multivariable Prediction Model for Individual Prognosis or Diagnosis (TRIPOD) statement. Future studies should aim to develop robust prediction models using larger, multi-ethnic cohorts—particularly those inclusive of Asian and African populations—employ rigorous methodological designs, and incorporate multi-center external validation to assess potential geographic and ethnic variations in PPM implantation risk factors.

## Data Availability

All data generated or analysed during this study are included in this published article [and its Supplementary Material]. Further inquiries can be directed to the corresponding author.

## Supplementary material

Supplementary Table S1

Lists the key items of PICOTS system.

Supplementary Table S2

Lists the complete list of search terms.

Supplementary Table S3

Lists the data extracted from the origin publications.

Supplementary Table S4

Lists the patients characteristics of development and validation cohorts in detail.

Supplementary Table S5

Lists the predictors included with each model in detail.

Supplementary Table S6

Summarizes the detailed surgical characteristics and procedural data extracted from the included studies.

Supplementary Table S7

Gives a detailed overview of the studies ROB, giving ROB and applicability for each domain.
