# Supplementary material for: Risk prediction models for permanent pacemaker implantation following transcatheter aortic valve replacement: a systematic review and meta-analysis
Source: Front Cardiovasc Med. 2025 Sep 25;12:1563597. doi: 10.3389/fcvm.2025.1563597 (PMC12507885; doi:10.3389/fcvm.2025.1563597)
Supplement: Supplementary file 1 [file Table1.docx]

**Supplemental Information**

**Supplementary Table 1** PICOTS

| Item | Criteria |
| --- | --- |
| Population | Patients after TAVR for aortic valve stenosis |
| Index prediction model | Risk prediction models for pacemaker implantation after TAVR that were developed and published (predictors ≥ 2) |
| Comparator | No competing model |
| Outcome | The occurrence of pacemaker implantation post-TAVR |
| Timing | The outcome was predicted after evaluating basic information at admission, electrocardiographic data, echocardiogram data, CTA data and TAVR procedural characteristics |
| Setting | The intended use of the risk prediction is to individualize the prediction of pacemaker implantation in patients undergoing TAVR, facilitating the implementation of preventive measures to prevent adverse events |

**Supplementary Table 2** Complete List of Search Terms

| Search Terms |
| --- |
| (“pacemaker*, artificial”[MESH] OR “artificial pacemaker*”[Title/Abstract] OR “cardiac pacemaker, artificial”[Title/Abstract] OR “artificial cardiac pacemaker*”[Title/Abstract] OR “cardiac pacemakers, artificial”[Title/Abstract] OR “pacemaker*, artificial cardiac”[Title/Abstract])  AND  (“Transcatheter Aortic Valve Replacement”[MESH] OR “Transcatheter Aortic Valve Implantation”[Title/Abstract])  AND  (“risk assessment”[MESH] OR “risk assessment”[Title/Abstract] OR predict*[Title/Abstract] OR “predictive model”[Title/Abstract] OR “predicting model”[Title/Abstract] OR “risk score”[Title/Abstract] OR “risk model”[Title/Abstract] OR “prognostic model”[Title/Abstract]) OR “risk prediction”[Title/Abstract] OR “risk factors*”[Title/Abstract] |

MESH, Medical Subject Headings; TAVR, transcatheter aortic valve replacement
